# Pityriasis rosea Gibert triggered by SARS-CoV-2 infection: A case report

**DOI:** 10.1097/MD.0000000000025352

**Published:** 2021-04-09

**Authors:** Victoria Birlutiu, Rares Mircea Birlutiu, Gabriela Mariana Iancu

**Affiliations:** a Lucian Blaga University of Sibiu, Faculty of Medicine Sibiu, Academic Emergency Hospital Sibiu - Infectious Diseases Clinic, Sibiu; b Lucian Blaga University of Sibiu, Faculty of Medicine Sibiu, FOISOR Clinical Hospital of Orthopedics, Traumatology, and Osteoarticular TB Bucharest; c Lucian Blaga University of Sibiu, Faculty of Medicine Sibiu, Academic Emergency Hospital Sibiu - Dermatology Clinic, Sibiu, Romania.

**Keywords:** coronavirus disease 2019, pityriasis rosea Gibert, severe acute respiratory syndrome coronavirus 2

## Abstract

**Rationale:**

Pityriasis rosea Gibert is an erythematous-papulosquamous dermatosis that frequently occurs in young adults. The etiopathogenesis of PR is still unknown, but is frequently associated with episodes of upper respiratory tract infections. It is likely that a new viral trigger of pityriasis rosea is the severe acute respiratory syndrome coronavirus 2 (SARS-CoV-2).

**Patient concerns:**

We present the case of a female patient in whom the diagnosis of pityriasis rosea led to the investigation and diagnosis of the SARS-CoV-2 infection. The patient presented to the Department of Dermatology for a 3 week duration of an extremely pruritic erythematous-squamous lesion, initially on the trunk and upper limbs, with extension to the lower limbs in the last week and the lesion respected the cephalic extremity, palms, and soles. One week before the rash, respiratory tract infection symptomatology was observed by the patient. At home, she underwent systemic treatment with antihistamines and topical medication with dermatocorticosteroids. The evolution was unfavorable, with the spread of the lesions and the accentuation of the pruritus.

**Diagnoses:**

Considering the actual epidemiological context, we performed a real-time reverse transcriptase-polymerase chain reaction (RT-PCR) assay from nasal and pharyngeal swabs for coronavirus disease 2019 (COVID-19) to investigate the PR etiology. The patient had a positive RT-PCR result, and was confirmed with SARS-CoV-2 infection.

**Interventions:**

Treatment was initiated with systemic corticosteroid therapy - hydrocortisone hemisuccinate 200 mg/day for 7 days, and loratadine 10 mg 2 times a day. Also, topical medication with dermatocorticosteroids and emollients was associated.

**Outcome:**

Under the treatment that was initiated a partial remission of the lesions after 7 days was observed.

**Lessons:**

Our reported case adds to the other findings regarding the association of PR with SARS-CoV-2 infection, in the context of the pandemic, suggesting the need to test patients with PR skin lesions for SARS-CoV-2 infection.

## 1 Introduction

Pityriasis rosea Gibert (PR) is an erythematous-papulosquamous dermatosis that frequently occurs in young adults, with a self-limited evolution that remits spontaneously within 2 months. The clinical aspect is highly suggestive for the diagnosis: a herald patch initially, with erythematous and scaly aspect, frequently located on the thorax and multiple oval erythematous-papulosquamous lesions, with a linear pattern, and a centrifugal progressive extension.

The etiopathogenesis of PR is still unknown, but the frequent association with episodes of upper respiratory tract infections before the onset of the disease, most likely, highlights the viral etiology of this dermatological condition. Cases of PR induced by some drugs and vaccines have been reported in the literature.^[1]^ Neoh et al analyzed the inflammatory infiltrate in the PR lesions and found that there is a deficiency of the natural killer cells and B lymphocytes at the lesion level. It also showed an increase in cluster of differentiation 4 T lymphocytes and Langerhans cells in response to the presence of a viral antigen.^[2]^ Broccolo et al demonstrated, using RT-PCR, that the reactivation of human herpesvirus 7 and 6 infections (HHV-7, HHV-6) plays an important role in the etiopathogenesis of PR.^[3]^

It seems that a new viral trigger of pityriasis rosea is the SARS-CoV-2. To date, 8 cases of pityriasis rosea in COVID-19 patients have been published in the literature. We present the case of a female patient in whom the diagnosis of pityriasis rosea led to the investigation and diagnosis of the SARS-CoV-2 infection.

## 2 Case report

We present the case of a 54-year-old Caucasian female, who presented to the Department of Dermatology, for a 3 week duration of an extremely pruritic erythematous-squamous lesion, initially on the trunk and upper limbs, that extended to the lower limbs in the last week and spared the cephalic extremity, palms and soles.

One week before the rash, the patient reported respiratory tract infection symptomatology (nasal obstruction, dysphagia, dry cough, and retrosternal pain). She was under treatment with antitussive drugs and antipyretics drugs (paracetamol 500 mg as needed), with a rapidly favorable evolution of respiratory manifestations. During the 3 weeks, until her examination in the Department of Dermatology, the patient underwent systemic treatment with antihistamines and topical medication with dermatocorticosteroids at home, under which the evolution was unfavorable, with the spread of the lesions and the accentuation of the pruritus. During these weeks the patient did not address herself to any medical specialist or her general practitioner. Considering the actual epidemiological context, the timeline, and the presented clinical data, we performed a real-time reverse transcriptase-polymerase chain reaction (RT-PCR) assay from nasal and pharyngeal swabs for COVID-19 to investigate the PR etiology. The patient had a positive RT-PCR result, being confirmed with SARS-CoV-2 infection. Therefore, the patient was transferred to the Department of Infectious Diseases for further investigations and treatment.

**Figure 1:**
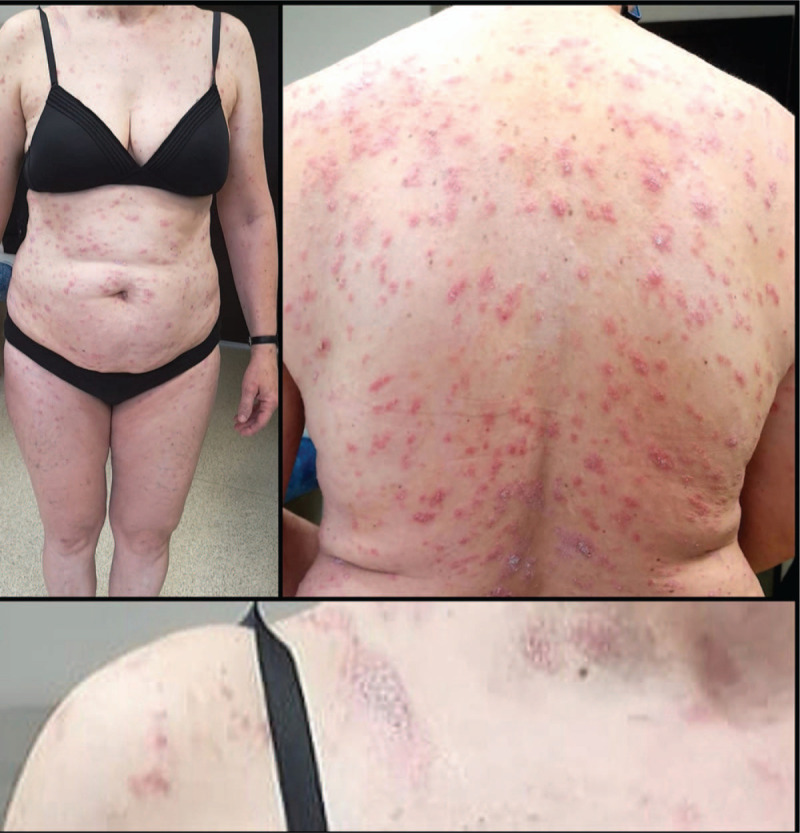
Disseminated erythematous-papulosquamous lesions, with right laterocervical herald patch (clinical aspect on admission).

At the time of admission on physical examination, the following were noticed: 36.5°C, peripheral oxygen saturation 98%, pulmonary examination without crackles on auscultation, respiratory rate of 16 breaths per minute, heart rate of 84 beats per minute, blood pressure 134 over 79 mm Hg. On dermatological examination, an eruption in the right supraclavicular area consisting of an oval erythematous squamous plaque, well delimited, of 2.5/1.5 cm, covered with white, adherent scales (herald patch) was observed. Disseminated oval erythematous-papulosquamous lesions, well delimited, with regular contour, with diameters of about 0.5 to 1 cm, with linear pattern, associated with pruritus, and with a centrifugal progressive extension (Fig. 1) were observed. The eruption did not involve the soles, palms, and facial area.

In our case, the clinical aspect was highly suggestive for PR. The patient presented all essential clinical features (oval erythematous-squamous lesions, with scaly aspect, with centrifugal progressive extension), all optional clinical features (supraclavicular heraldic plaque before the eruption; lesions with a linear pattern, located on the thorax, abdomen, and on the upper and lower limbs, less than 10% of them being located towards the extremities) and without any exclusive clinical features (without vesicular aspect, without the involvement of the soles, palms, and facial area, and with negative serology for syphilis).^[4]^

Laboratory examinations, respectively complete blood count, C-reactive protein, fibrinogen, syphilis serology, procalcitonin, ferritin, D Dimers were between normal values, except the erythrocyte sedimentation rate 23 mm/hours (reference values 0–20 mm/hours) and Lactate dehydrogenase 247 U/L (reference values 125–220 U/L). The pulmonary radiography revealed no pathological changes (A pulmonary radiography is mandatory to be performed for each SARS-CoV-2 diagnosed patient according to the national guidelines as being one of the minimum requested investigations).

To rule out a secondary syphilis or an HIV infection, we performed luetic serology and HIV antibodies test, both being negative. The clinical appearance and the lack of medication use with a possible toxidermic role, allowed us to exclude secondary drug eruptions. The clinical aspect allowed us to differentiate PR from other dermatoses: lichen ruber planus, psoriasis associated with gouty arthritis, parapsoriasis, erythema multiforme, or tinea corporis. To exclude other causes of viral exanthema we conducted the following serological tests (IgM for parvovirus B19, HHV-6, HHV-7, and Epstein Barr virus) which were negative.

**Figure 2:**
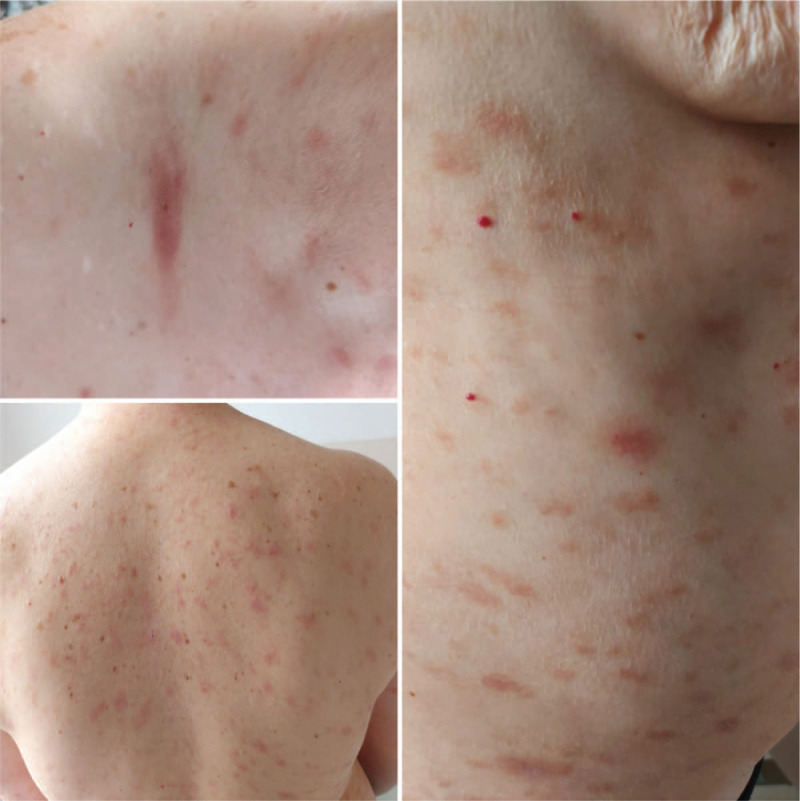
Clinical aspect of the lesions after 7 days of treatment.

Given the persistence and progressive nature of the lesion during the 3 weeks, with associated pruritus and with an inflammatory skin appearance, treatment was initiated with systemic corticosteroid therapy - hydrocortisone hemisuccinate 200 mg/day for 7 days, and loratadine 10 mg 2 times a day. Topical medication with dermatocorticosteroids and emollients were used with partial remission of the lesions after 7 days (Fig. 2). Two weeks after the admission into the Infectious Diseases Department, the patient was discharged with partial resolution of the skin lesions, without respiratory manifestations, and with a negative SARS-CoV-2 RT-PCR from nasal and pharyngeal swabs.

## 3 Discussions

Coronavirus disease 2019 (COVID-19) is an infectious disease with multi-organ involvement, including the skin. Mainly, the cutaneous manifestations appear due to a large influx of proinflammatory cytokines at the skin level and/or due to some processes of microthrombosis at the dermal level. In the dynamics of COVID-19 cases, Recalcati S described a significant percentage of hospitalized patients with SARS-CoV-2 infection (20.4%) who developed cutaneous manifestations at the onset of the disease (9.09%) or after discharge from the hospital (11.31%),^[5]^ while Guan et al. identified skin lesions in 0.2% of 1099 patients with COVID-19.^[6]^

The lesion associated with SARS-CoV-2 infection is extremely varied, from erythematous papular rash, urticarial, chickenpox-like vesicles, erythema multiforme-like rash to purpuric rash, necrotic/livedo reticularis, and chilblain-like lesions.^[7,8]^

Pityriasis rosea is being recognized as a manifestation of the reactivation of human herpesvirus 7 and 6 infection.^[3,9,10]^ Dursun et al report a fivefold increase in the number of cases of pityriasis rosea during the SARS-CoV-2 pandemic, suggesting either the reactivation of the human herpesvirus 6 infection during COVID-19 infection or psycho-emotional factors were related to the passage through infection.^[11]^

In May 2020, Ehsani et al reported the first case of PR associated with SARS-CoV-2 infection, the case of a 27-year-old male patient on admission being subfebrile, with associatedasthenia and digestive and respiratory manifestations - imaging studies suggestive for SARS-CoV-2 pneumonia with favorable evolution under treatment with antihistamines and antipyretics drugs, and topical corticosteroids.^[1]^

Another case report of pityriasis rosea associated with SARS-CoV-2 infection belongs to David Martín Enguix et al which describes the case of a 19-year-old female with asymptomatic SARS-CoV-2 infection, the appearance of the characteristic skin lesions, round-oval, erythematous-squamous lesions, on the trunk and upper limbs, without herald patch. Topical therapy with dermatocorticosteroids and emollients allowed almost complete remission of lesions within 1 month after the initial diagnosis.^[12]^

Veraldi et al described 2 cases of PR, in young male patients, with minor digestive or general manifestations, without respiratory impairment, with a favorable outcome in 3 weeks under systemic antihistamine and topical corticosteroids therapy.^[13]^

Another case of PR, preceded by respiratory manifestations, is the case of a 26-year-old female patient, that was also described by Merhy et al. In this case report, the authors considered that PR had as a trigger factor the COVID-19, either a possible reactivation of a latent infection with human herpesvirus 6 or 7 or Epstein-Barr virus infection, without having performed any virological studies.^[14]^

Johansen et al reported in the literature 2 cases of PR in female patients with phototype III and IV, with asymptomatic forms of COVID-19, in which the cutaneous manifestations preceded the diagnosis of SARS-CoV-2 infection.^[15]^

The association of pityriasis rosea at a 12-year-old boy, 4 weeks after passing through SARS-CoV-2 infection, has also been described in the pediatric literature, suggesting an immune mechanism induced by the SARS-CoV-2 virus.^[16]^

The PR cases associated with SARS-CoV-2 infection published so far are at young patients (12–39 years-old), with a mean age of 24.12 years, with equal distribution by gender (50% male patients). The time period between the onset of the rash and the presentation of the patient to the doctor varied between 3 days and 2 weeks, the rash is mainly associated with pruritus (75%). Four of the 8 patients were symptomatic patients, with general, respiratory and digestive manifestations, having mild forms of COVID-19. Laboratory investigations were within normal limits, except for 1 case of SARS-CoV-2 pneumonia that also was associated with a biological inflammatory syndrome. The used therapeutic management was specified in 6 of the 8 cases: antihistamines drugs (50%), antipyretics drugs (16.67%), and topical corticosteroids (100%). Healing of the skin lesions required 2 to 4 weeks.

Our presented case of PR is the first case associated with SARS-CoV-2 infection in a 54-year-old patient, who presented herself late to the specialist (after 3 weeks from the onset of the skin lesion) and required systemic corticosteroid therapy to control PR.

The role of SARS-CoV-2 as a trigger in the onset of PR is suggested in all case reports from the literature, but the serological exclusion of the concomitant involvement of other viruses has not been achieved. The onset of cutaneous manifestations 1 week after the neglected respiratory episode and the RT-PCR positivity for SARS-CoV-2 at 4 weeks after the onset of respiratory manifestations suggest that SARS-CoV-2 infection was the main trigger of the rash, either directly by a solitary action, either indirectly by inducing an important inflammatory response - hyperactivity of proinflammatory cytokines (especially Interleukin 1β)^[17]^ or a possible reactivation of HHV-6 or HHV-7.

### 3.1 Informed consent

Written informed consent was obtained from the patient for publication of their case report and any accompanying images. The study was accepted by the Ethics Committee of the hospital and they encouraged publishing the article. A copy of the written consent is available for review by the Editor-in-Chief of this journal.

## 4 Conclusions

Our reported case adds himself to the other findings regarding the association of PR with SARS-CoV-2 infection, in the context of the pandemic, suggesting the need to test patients with PR skin lesions for SARS-CoV-2 infection.

## Author contributions

All authors contributed equally to this manuscript in terms of acquisition, analysis, and interpretation of data, conception, and design, drafting the manuscript. All authors read and approved the final manuscript.

**Conceptualization:** Victoria Birlutiu, Rares Mircea Birlutiu, Gabriela Mariana Iancu.

**Data curation:** Victoria Birlutiu.

**Formal analysis:** Victoria Birlutiu, Rares Mircea Birlutiu, Gabriela Mariana Iancu.

**Investigation:** Victoria Birlutiu.

**Methodology:** Victoria Birlutiu.

**Supervision:** Victoria Birlutiu, Gabriela Mariana Iancu.

**Validation:** Victoria Birlutiu, Gabriela Mariana Iancu.

**Visualization:** Victoria Birlutiu, Rares Mircea Birlutiu, Gabriela Mariana Iancu.

**Writing – original draft:** Victoria Birlutiu, Gabriela Mariana Iancu.

**Writing – review & editing:** Rares Mircea Birlutiu, Gabriela Mariana Iancu.
